# Nidogen 1-enriched extracellular vesicles drive HCC progression by inducing mregDC differentiation and Treg infiltration

**DOI:** 10.20517/evcna.2026.03

**Published:** 2026-09-08

**Authors:** Lu Xia, Tingmao Xue, Yining Xu, Judy Wai Ping Yam, Xiaowen Mao

**Affiliations:** ^1^State Key Laboratory of Mechanism and Quality of Chinese Medicine, Institute of Chinese Medical Sciences, University of Macau, Macao 999078, China.; ^2^Department of Pathology, Li Ka Shing Faculty of Medicine, The University of Hong Kong, Hong Kong SAR, China.; ^3^State Key Laboratory of Liver Research (The University of Hong Kong), Hong Kong SAR, China.; ^4^Materials Innovation Institute for Life Sciences and Energy (MILES), HKU-SIRI, Shenzhen 518000, Guangdong, China.; ^#^These authors contributed equally to this work.

**Keywords:** NID1, extracellular vesicles, dendritic cells, Tregs, hepatocellular carcinoma

## Abstract

**Aim:** Nidogen 1 (NID1) is a secreted pro-tumorigenic protein. While extracellular vesicle (EV)-NID1 is known to accelerate hepatocellular carcinoma (HCC) progression, its role in reshaping the HCC immune microenvironment remains undefined. This study investigated how EV-NID1 modulates dendritic cells (DCs) to establish an immunosuppressive niche that drives liver cancer growth.

**Methods:** The TIMER3.0 database was used to assess the correlations of NID1 with DCs and regulatory T cells (Tregs). A Transwell co-culture system using bone marrow-derived DCs was employed to evaluate the paracrine effects of HCC cells. EVs were isolated by differential ultracentrifugation, and their identity and purity were verified. Orthotopic and subcutaneous HCC mouse models were established to assess immune cell infiltration *in vivo*. DC adoptive transfer and Treg depletion assays were conducted to elucidate the underlying mechanism.

**Results:** High NID1 expression in HCC was associated with increased infiltration of myeloid DCs and Tregs, and their co-occurrence predicted poor patient survival. *In vitro*, EV-NID1 induced DC maturation and promoted their polarization toward mature DCs enriched in immunoregulatory molecules (mregDC), accompanied by elevated secretion of C-C chemokine ligand 17 (CCL17). These polarized DCs promoted CD4^+^ T cell proliferation and FOXP3^+^ Treg formation. *In vivo*, NID1-high tumors exhibited accelerated growth and increased infiltration of mregDCs and Tregs in tumors, lymph nodes, and spleens. NID1 knockdown reduced tumor growth, whereas adoptive transfer of EV-NID1-pulsed DCs partially rescued tumor progression. Furthermore, Treg depletion markedly suppressed the growth of NID1-high tumors.

**Conclusions:** This study suggests that NID1-enriched EVs derived from HCC promote tumor progression by inducing a regulatory phenotype in DCs and the expansion of Tregs.

## INTRODUCTION

Liver cancer constitutes a significant global health burden characterized by high mortality and rising incidence^[1]^. It ranks as the sixth most common cancer and the third leading cause of cancer mortality worldwide^[2]^. Hepatocellular carcinoma (HCC) is the predominant form of liver cancer, representing approximately 75%-85% of all cases^[3]^. Achieving a cure is the ultimate goal in HCC therapy, yet current treatment options are still limited to palliative care. Therefore, there is an urgent need to further elucidate the pathogenesis of liver cancer and to identify new therapeutic targets^[4]^.

Extracellular vesicles (EVs) are membrane-bound particles released by cells under both physiological and pathological conditions^[5]^. The diverse functional properties of EVs stem from their unique composition of lipids, proteins, and RNAs, enabling a wide range of potential functions^[6]^. EVs drive tumor progression, including growth, metastasis, and drug resistance, through the transfer of oncogenic cargo and their pro-angiogenic and immunosuppressive effects^[7]^. The disruption of T cell, natural killer (NK) cell, and dendritic cell (DC) function by tumor-derived EVs mediates tumor immune evasion^[8,9]^. EVs carrying programmed cell death ligand 1 (PD-L1) exploit the PD-1/PD-L1 axis to facilitate tumor immune evasion, directly interacting with and inhibiting anti-tumor immune cells^[10]^. Moreover, tumor-derived EVs orchestrate the formation of a pre-metastatic niche and an immunosuppressive environment, ultimately facilitating tumor immune evasion^[11]^. Paradoxically, EVs can also mediate anti-tumor responses by presenting tumor antigens to activate T cells or by delivering stress proteins such as heat shock protein 70 (HSP70) to stimulate NK cells, thereby countering tumor progression^[12]^. These findings underscore the functional duality of tumor-derived EVs in shaping the tumor immune microenvironment.

As an integral component of the basement membrane and extracellular matrix (ECM), nidogen 1 (NID1) serves to connect other constituent proteins, such as collagen, fibronectin, and laminin^[13]^. NID1 has been implicated in a variety of human cancers^[14,15]^. In addition to being a structural component, NID1 is actively released into the extracellular environment either as a soluble secretory protein or encapsulated within EVs^[16]^. Consistently elevated NID1 levels have been found in the secretome of lung metastatic breast cancer, colorectal tissues, and melanoma cells^[17,18]^. HCC-derived EVs enriched in NID1 activate pulmonary fibroblasts, thereby promoting the formation of a supportive niche for the colonization of disseminated tumor cells in the lungs^[19]^. While substantial evidence has implicated NID1 in cancer pathogenesis, a significant gap remains in understanding its role in tumor immunomodulation.

To address these gaps, the present study uncovers a previously unreported local pro-tumorigenic role of NID1 associated with EVs (EV-NID1) within primary HCC tissue. Using *in vitro* and *in vivo* animal models, we demonstrate that HCC-derived EV-NID1 rewires DC homeostasis to drive the formation of immunosuppressive mature DCs enriched in immunoregulatory molecules (mregDCs). These mregDCs further secrete C-C motif chemokine ligand 17 (CCL17), thereby recruiting tumor-promoting regulatory T cells (Tregs) and ultimately establishing an intratumoral immunosuppressive microenvironment that promotes primary HCC growth. Notably, to our knowledge, this study provides the first functional characterization of EV-NID1-mediated immune suppression in primary liver tumors.

## METHODS

### Cell culture

Mouse HCC cell line Hepa1-6 was purchased from American Type Culture Collection (ATCC) and cultured according to the ATCC recommendations. Murine p53^-/-^; c-Myc hepatoblast cell line 1753^p53-/-^cmyc was provided by Scott Lowe (Memorial Sloan Kettering Cancer Center, New York)^[20]^. This cell line was cultured according to the provider’s recommendations. All cell lines underwent routine testing for mycoplasma before use in experiments. All experiments were performed using cells between passages 10 and 15.

### CD4^+^ T cell and bone marrow-derived DC (BMDC) isolation

Bone marrow progenitors from mouse femurs and tibias were cultured in 10-cm dishes at 2 × 10^6^ cells per dish in BMDC medium [RPMI 1640 with fetal bovine serum (FBS)], penicillin/streptomycin; all from Gibco supplemented with 20 ng/mL granulocyte-macrophage colony-stimulating factor (GM-CSF) and 10 ng/mL interleukin-4 (IL-4) (Peprotech). On day 3, an additional 10 mL of BMDC medium was added to the dish, and on day 6, half of the medium was replaced with fresh medium. On day 8, nonadherent and loosely adherent cells were mainly immature BMDCs (iDCs) ready for use. CD4^+^ T cells were isolated using microbeads according to the manufacturer’s instructions.

### Isolation of EVs from conditioned cell culture medium

For EV isolation, cells were grown in medium with 10% FBS that had been depleted of EVs by overnight centrifugation at 100,000 × *g*, 4 °C (Beckman XPN100). After culturing cells in this medium for 72 h, supernatants were collected, and EVs were purified by differential centrifugation, beginning with a 3,000 × *g* spin for 15 min to eliminate cell debris and dead cells (Eppendorf 5920R). After centrifugation at 20,000 × *g* for 30 min at 4 °C (Beckman XPN100) to discard microvesicles, the supernatants were filtered (0.22 µm, Millipore) and ultracentrifuged at 100,000 × *g* for 2 h at 4 °C to pellet EVs. The pelleted EVs were washed with phosphate-buffered saline (PBS) and re-collected by ultracentrifugation under identical conditions.

### Validation of isolated EVs

Proteins were extracted from isolated EVs and analyzed via Western blotting using anti-Alix (Cell Signaling Technology), anti-130 kDa cis-Golgi matrix protein 1 (GM130) (Abcam), anti-p62 (Abcam), and anti-β-actin (Sigma-Aldrich) antibodies. For integrity assessment, purified EVs suspended in PBS were placed onto Formvar-carbon-coated nickel grids. The grids were then stained with 2% uranyl acetate, air-dried, and imaged using a Philips CM100 transmission electron microscope (TEM) (FEI Company). For size range measurement, EVs were analyzed using a ZetaView BASIC nanoparticle tracking analyzer (NTA) PMX-120 (Particles Metrix GmbH).

### Establishment of NID1 stable knockdown and overexpression cell lines

NID1 stable knockdown (KD) clones were generated using the pLKO.1 shRNA plasmid (Addgene) with target sequence GCCAATTATCTTGTCTGTATA (5’→3’). On-target normal control (NC) clones (shCTL) were generated using the pLKO.1-hygro plasmid with target sequence CAACAAGATGAAGAGCACCAA (5’→3’). For NID1 stable overexpression (OE) clones, the full-length murine *Nid1* coding sequence was subcloned into the pLVX-Puro lentiviral expression vector. The MISSION® lentiviral packaging mix and Fugene® 6 transfection reagent (Promega) were employed to transfect the shNID1 plasmid into HEK293FT cells. After 24 h, viral supernatant was centrifuged and filtered through a 0.45-μm filter. Polybrene transduction enhancer together with the viral supernatant was then added to seeded cells. Forty-eight hours after transduction, cells were selected with hygromycin B and puromycin (InvivoGene).

### Flow cytometry

Fluorochrome-conjugated anti-mouse antibodies for CD11c, CD80, and CD86 were obtained from Invitrogen; antibodies against CD40, CD45, T cell receptor (TCR), CD4, CD8, C-C motif chemokine receptor type 7 (CCR7), PD-L1, PD-L2, and T-cell immunoglobulin and mucin domain-containing protein 3 (TIM-3), Foxp3, CD200 were obtained from BioLegend; anti-mouse major histocompatibility complex class II (MHC II) was obtained from eBioscience. All antibody dilution ratios were 1:200. Cell proliferation was quantified with a CellTrace^TM^ Violet cell proliferation kit (Thermo Fisher). eBioscience Foxp3/Transcription Factor Staining Buffer Set (Invitrogen) was used for Foxp3 staining. Briefly, the Fix/Permeabilization Concentrate is diluted with Fixation/Perm Diluent at a ratio of 1:3, and then added to the cell samples. The samples are incubated overnight at 4 °C. On the next day, the cells are washed and incubated with Permeabilization Buffer diluted with ultrapure water at a ratio of 1:9. The resulting cell samples are then ready for subsequent staining. Stained cells or samples underwent flow cytometric analysis on an LSR Fortessa (BD Biosciences). Flow cytometry data were analyzed with FlowJo v10.0 (Treestar).

### Western blotting

Cell lysis was performed in radio-immunoprecipitation assay (RIPA) buffer (Beyotime) containing protease and phosphatase inhibitor cocktails (Roche). Proteins were separated using sodium dodecyl sulfate-polyacrylamide gel electrophoresis (SDS-PAGE) and blotted onto a polyvinylidene fluoride (PVDF) membrane. Membranes were blocked with 5% skimmed milk, probed with primary antibodies (1:1,000), and then incubated with horseradish peroxidase (HRP)-conjugated secondary antibodies (1:5,000), HRP-linked anti-rabbit IgG (Abcam), or HRP-linked anti-mouse IgG (Abcam). The target proteins were detected using enhanced chemiluminescence (ECL) reagent (Cytiva) and a ChemiDoc MP imaging system (Bio-Rad). Western blot analysis in all figures was performed with three biological replicates.

### Enzyme-linked immunosorbent assay (ELISA)

Serum NID1 levels in C57BL/6J mice were quantified using a mouse NID1 ELISA kit (WELLBIO). A mouse CCL17 ELISA kit (JIANGLAI) was used to measure the concentration of CCL17 in BMDC culture supernatants and in serum from C57BL/6J mice. A mouse interleukin-10 (IL-10) ELISA kit (JIANGLAI) was used to determine serum IL-10 levels in C57BL/6J mice. All ELISA experiments were performed according to the manufacturer’s instructions.

### Colony formation assay

Assessment of clonogenic survival was achieved by means of a colony formation assay. Briefly, single-cell suspensions were plated in 6-well plates at 800 cells/well. The cells were then cultured for 10-14 days in complete medium, with the medium being refreshed every 5-7 days. Subsequently, the resulting colonies were fixed with 4% paraformaldehyde (Biosharp), stained with 0.5% crystal violet (Sigma-Aldrich), and manually counted. Only colonies containing more than 50 cells were included in the analysis.

### Cell migration assay

For the cell migration assay, 3 × 10^4^ cells in serum-free medium were seeded in the upper chamber of Transwells. Dulbecco’s modified Eagle medium (DMEM) (Gibco) supplemented with 10% FBS was added to the bottom chamber as a chemoattractant. Following a 24- to 48-h incubation, cells were fixed with methanol for 40 min and subsequently stained with crystal violet for 20 min. Four visual fields were randomly acquired from each well, and the counts of migrated cells were determined.

### Animal studies

Experimental procedures involving animals were carried out in compliance with research protocol UMARE-0303-2024, approved by the Animal Ethics Committee of the University of Macau. All mice used in the experiments were obtained from and maintained in a specific pathogen-free (SPF) animal facility at the University of Macau.

### Orthotopic liver implantation model

An orthotopic liver implantation model was established using C57BL/6J mice aged 6-8 weeks to evaluate the effects of NID1 on liver cancer growth and tumor immune infiltration. Briefly, 5 × 10^4^ 1753^p53-/-^cmyc NID1 NC or NID1 KD cells were suspended in 20 µL of PBS and injected into the left lobe of the liver. Mice were sacrificed at 4 weeks post-intrahepatic injection, and the tumor, tumor-draining lymph nodes (TdLN) (celiac and portal), and spleen were used for experiments.

### Subcutaneous injection model

1753^p53-/-^cmyc NID1 NC or NID1 KD cells were administered subcutaneously into the right flank of C57BL/6J mice at the concentration of 5 × 10^4^ in 100 μL. Tumor growth was assessed at regular intervals by measuring both length and width with a caliper. Tumor volumes were calculated by the formula V (mm^3^) = L (mm) × S^2^ (mm^2^)/2, where L and S represent the longest and shortest perpendicular tumor diameters, respectively. All mice were euthanized upon experiment completion, and tumors were resected for measurement and later use.

### Adoptive transfer of DCs

Male C57BL/6J mice (6-8 weeks old) were randomized into four groups (NID1 NC, NID1 KD + PBS, NID1 KD + NC-EV-pulsed DC, NID1 KD + KD-EV-pulsed DC) (*n* = 5). On day 0, 4 × 10^5^ 1753^p53-/-^cmyc NID1 NC or NID1 KD cells were subcutaneously injected into mice. On days 7, 14, and 21, mice in the NID1 KD + PBS, NID1 KD + NC-EV-pulsed DC, and NID1 KD + KD-EV-pulsed DC groups were administered via the tail vein with PBS, NC-EV-pulsed DC (10^6^ cells/mouse), and KD-EV-pulsed DC (10^6^ cells/mouse), respectively. Tumor sizes were recorded every 7 days, and on day 28, mice were sacrificed, and tumors were harvested for further analysis. Flow cytometry analysis of intratumoral Treg populations was performed using *n* = 3 evaluable samples per group.

### Treg depletion assay

Male C57BL/6J mice (6-8 weeks old) were randomized into three groups (NID1 NC + IgG, NID1 NC + Treg Ab, and NID1 KD) (*n* = 5). Three days after the subcutaneous injection of 1753p53^-/-^cmyc NID1 NC and NID1 KD cells, the NID1 NC + IgG and NID1 NC + Treg Ab groups received respective injections of IgG or anti-Treg antibody (500 μg/mouse). Tumor sizes were recorded every 7 days, and on day 28, mice were sacrificed, and tumors were harvested for further analysis.

### Database mining

TIMER3.0 (https://compbio.cn/timer3/) was used to quantify immune cell infiltration. The MCPCOUNTER and QUANTISEQ algorithms were applied with canonical gene signature sets from the built-in reference matrix: myeloid DCs (mDCs) were defined by integrin alpha-X (ITGAX), HLA class II histocompatibility antigen, DR alpha chain (HLA-DRA), CD1C, high-affinity IgE receptor alpha subunit (FCER1A) and C-type lectin domain family 10 member A (CLEC10A); Tregs were defined by forkhead box protein P3 (FOXP3), interleukin-2 receptor subunit alpha (IL2RA), cytotoxic T-lymphocyte-associated protein 4 (CTLA4) and T cell immunoreceptor with Ig and ITIM domains (TIGIT). Spearman’s rank correlation analysis was performed with tumor purity adjustment. A positive correlation was indicated by r > 0, and statistical significance was set at *P* < 0.05.

For survival analysis stratified by NID1 expression and Treg infiltration, immune cell scores were calculated via the CIBERSORT algorithm. After Z-score transformation, Cox proportional hazards regression models were constructed, with *P* < 0.05 regarded as statistically significant.

### Statistical analysis

Statistical analyses were conducted with GraphPad Prism 10.0 (La Jolla, CA, USA). Results are presented as mean ± standard deviation (SD), and Student’s *t*-test was employed to assess differences between two groups. One-way ANOVA with a post hoc test (Tukey’s test) was adopted for intergroup comparisons across multiple groups. Before applying parametric tests, the normality of the data was assessed. *P* < 0.05 was considered statistically significant.

## RESULTS

### NID1 is highly expressed in HCC patients and promotes the maturation of DCs

Our previous reports have shown that NID1 is highly expressed in HCC patients^[16,19]^. To further elucidate the intrinsic link between NID1 and tumor immune infiltration, the TIMER3.0 database was interrogated, and the analysis revealed that NID1 is highly expressed in HCC, and patients with high NID1 expression had significantly reduced overall survival [Figure 1A]. In addition, NID1 showed positive correlations with mDC and Treg infiltration [Figure 1B and C]. Notably, patients exhibiting both high NID1 expression and high Treg infiltration demonstrated a significantly poorer overall survival (*n* = 371) [Figure 1C]. These results suggest that NID1 may play a potential regulatory role in modulating DC and Treg infiltration within the HCC tumor microenvironment.

**Figure 1:**
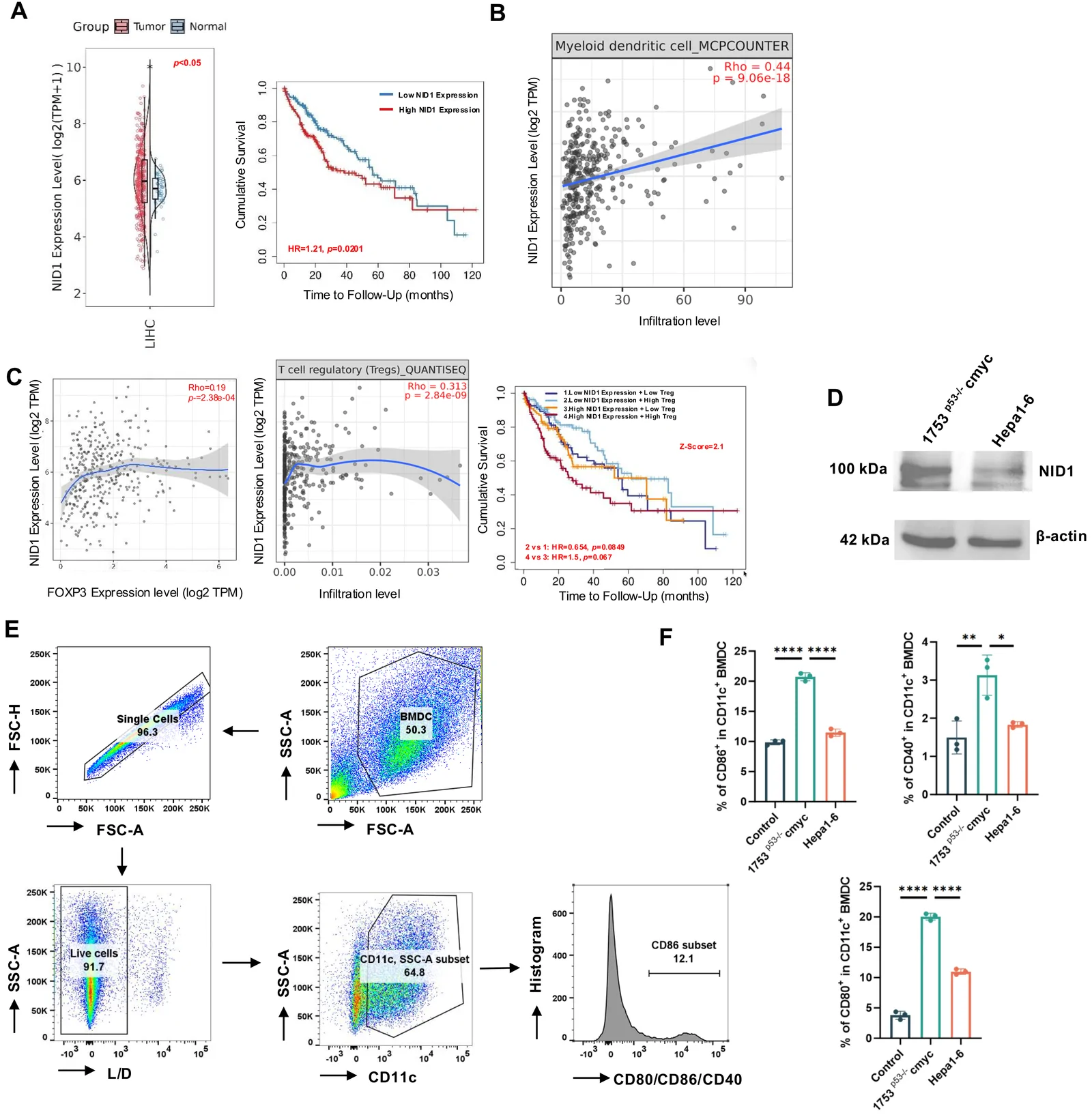
NID1 is highly expressed in HCC patients and promotes DC maturation. (A) The TIMER3.0 database shows NID1 expression in liver cancer and normal tissues, as well as its prognostic impact on patients with liver cancer; (B) Analysis of the relationship between NID1 and mDC infiltration in liver cancer tissues using the TIMER3.0 database; (C) The TIMER3.0 database shows a correlation between NID1 and Treg infiltration in liver cancer, with their combined impact on patient survival; (D) Western blot of NID1 expression in 1753^p53-/-^cmyc and Hepa1-6 cells; (E) Gating strategy for flow cytometry analysis of the expression changes in BMDC maturation markers; (F) Flow cytometry analysis of changes in expression of CD80, CD86, and CD40 in BMDCs after co-culture with 1753^p53-/-^cmyc and Hepa1-6 cells using a Transwell system. Data are presented as mean ± SD, with statistical significance determined by one-way ANOVA (Tukey’s test). Biological replicates are represented as individual data points. Western blot analysis was performed with three biological replicates. ^*^P < 0.05; ^**^P < 0.01, ^****^P < 0.0001. NID1: Nidogen 1; HCC: hepatocellular carcinoma; DC: dendritic cell; mDC: myeloid dendritic cell; Treg: regulatory T cell; FOXP3: forkhead box protein P3; TPM: transcripts per million; HR: hazard ratio; BMDC: bone marrow-derived dendritic cell; TIMER3.0: Tumor Immune Estimation Resource 3.0; LIHC: liver hepatocellular carcinoma; MCPCOUNTER: Microenvironment Cell Populations-counter; QUANTISEQ: quantitative immune cell deconvolution method; FSC-A: forward scatter-area; FSC-H: forward scatter-height; SSC-A: side scatter-area; CD11c: cluster of differentiation 11c; CD80: cluster of differentiation 80; CD86: cluster of differentiation 86; CD40: cluster of differentiation 40; L/D: live/dead viability dye; SD: standard deviation; ANOVA: analysis of variance.

As professional antigen-presenting cells (APCs), DCs have a robust capacity for antigen processing and presentation^[21]^. DCs present processed antigens to naïve T cells, providing critical signals for activation and driving differentiation into either CD4^+^ or CD8^+^ T cells^[22]^. Given the pivotal “initiating” role of DCs in T cell activation, we hypothesized that NID1 may modulate Tregs via its influence on DCs, thereby fostering an immunosuppressive microenvironment that promotes the progression of HCC. To investigate the regulatory role of NID1 on DCs, murine HCC cell lines with high NID1 expression (1753^p53-/-^cmyc) and low NID1 expression (Hepa1-6) were selected [Figure 1D]. Due to the secretory nature of NID1^[16]^, immature DCs (iDCs) induced from BMDCs were co-cultured with either 1753^p53-/-^cmyc or Hepa1-6 cell lines using a Transwell system. We examined the expression of CD40, CD80, and CD86 on CD11c^+^ BMDCs following co-culture with 1753^p53-/-^cmyc /Hepa1-6 [Figure 1E]. The results demonstrated that co-culture with the high-NID1-expressing HCC cells significantly upregulated the expression of the DC maturation markers CD40 (*P* = 0.0167), CD80 (*P* < 0.0001), and CD86 (*P* < 0.0001) on the cell surface [Figure 1F]. This suggests that secretory NID1 can promote DC maturation.

### NID1-enriched EVs facilitate the maturation of DCs

NID1 can be released as a secretory protein or carried by EVs^[16]^. To distinguish whether the free protein or the EV-associated form of NID1 promotes DC maturation, iDCs were treated with murine recombinant NID1 (rNID1) protein. DC maturation was not induced by rNID1 at concentrations up to 10 μg/mL [Supplementary Figure 1], indicating that the free NID1 protein does not drive this process. Next, DCs were treated with different conditioned media: plain EV-depleted medium (fresh medium without EVs), NID1 EV-containing conditioned medium (conditioned medium from 1753^p53-/-^cmyc cells cultured in EV-depleted medium), and NID1 EV-depleted conditioned medium (supernatant of NID1 EV-containing conditioned medium after ultracentrifugation, no EVs present). A significant downregulation in the surface expression of maturation markers CD40 (*P* = 0.0175), CD80 (*P* = 0.0307), and MHC II (*P* = 0.0001) was observed specifically in DCs cultured with the EV-depleted conditioned medium [Figure 2A], indicating a critical role for EVs in DC maturation. To further investigate the impact of EV-NID1 on DCs, EVs from the conditioned medium of 1753^p53-/-^cmyc cells were isolated, and positive markers (Alix) and negative markers (GM130 and p62) were determined [Figure 2B]. Notably, NID1 was significantly enriched in the isolated EVs [Figure 2B]. TEM and NTA further confirmed the size and morphology of the isolated EVs [Figure 2C and D]. DCs were then treated with the isolated EVs at 10 μg/mL. The results suggested that EV treatment promoted increased expression of DC surface maturation markers CD40 (*P* = 0.0002) and MHC II (*P* = 0.0071) [Figure 2E]. Moreover, EV-treated DCs exhibited elevated levels of CCR7 (*P* = 0.0013), suggesting that NID1-enriched EVs can facilitate DC migration [Figure 2F]. Based on the association between NID1 expression and Treg infiltration in HCC [Figure 1C], the level of CCL17, a key chemokine for Treg recruitment, in the culture supernatant of DCs was determined. DCs cultured with NID1-enriched EVs tended to have increased CCL17 secretion [Figure 2G]. Collectively, these findings suggest that NID1-enriched EVs stimulate DC maturation and Treg recruitment during HCC progression.

**Figure 2:**
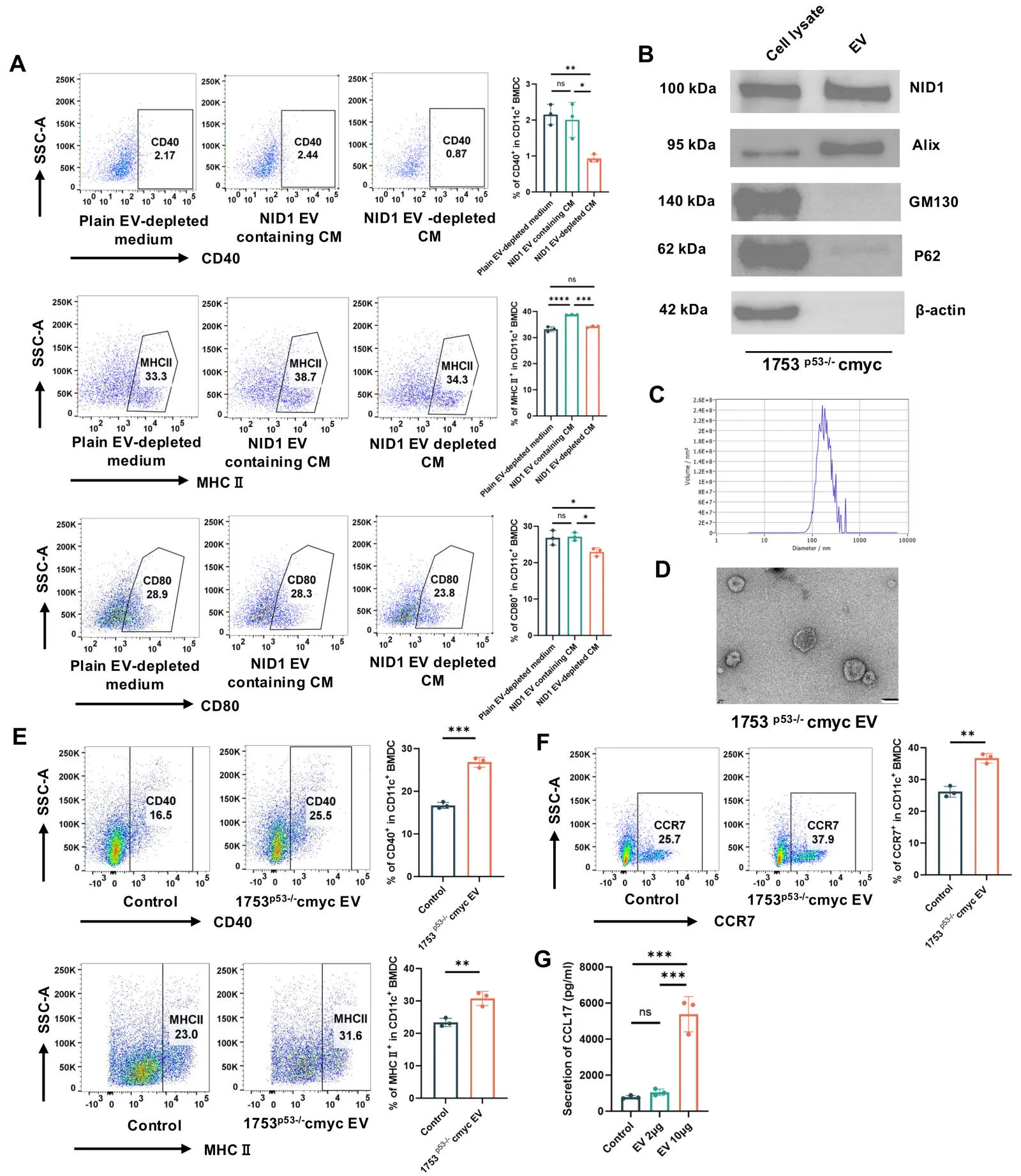
NID1-enriched EVs facilitate DC maturation. (A) Flow cytometry analysis of changes in the expression of CD40, CD80, and MHC II on BMDCs after treatment with conditioned media from 1753^p53-/-^cmyc with or without EVs; (B) Detection by Western blot of EV positive markers (Alix), negative markers (GM130, p62), and NID1 expression in the cell lysate and EVs collected from 1753^p53-/-^cmyc cells; (C) NTA profiles of EVs isolated from 1753^p53-/-^cmyc cells; (D) TEM imaging of EVs secreted by 1753^p53-/-^cmyc cells. Scale bar: 100 nm; (E) Flow cytometry analysis showed changes in the expression of CD40 and MHC II on BMDCs after treatment with EVs derived from 1753^p53-/-^cmyc; (F) Flow cytometry analysis of CCR7 expression on BMDCs after treatment with EVs derived from 1753^p53-/-^cmyc cells; (G) ELISA determination of CCL17 levels in BMDC culture supernatants after treatment with EVs derived from1753^p53-/-^cmyc cells at doses of 2 μg/mL and 10 μg/mL. Data are presented as the mean ± SD, with statistical significance determined by one-way ANOVA (multiple groups) (Tukey’s test) or Student’s *t*-test (two groups). Biological replicates are represented as individual data points. Western blot analysis was performed with three biological replicates. ^*^P < 0.05; ^**^P < 0.01, ^***^P < 0.001, ^****^P < 0.0001. NID1: Nidogen 1; EV: extracellular vesicle; DC: dendritic cell; BMDC: bone marrow-derived dendritic cell; CM: conditioned medium; MHC I: major histocompatibility complex class I; MHC II: major histocompatibility complex class II; CD40: cluster of differentiation 40; CD80: cluster of differentiation 80; CD11c: cluster of differentiation 11c; Alix: ALG-2-interacting protein X; NTA: nanoparticle tracking analysis; TEM: transmission electron microscopy; CCR7: C-C chemokine receptor 7; CCL17: C-C motif chemokine ligand 17; ELISA: enzyme-linked immunosorbent assay; SSC-A: side scatter-area; SD: standard deviation; ANOVA: analysis of variance; ns: not significant.

### EV-NID1 promotes the differentiation of DCs into mregDCs

To confirm the essential role of EV-NID1 in tumor immunomodulation, stable KD of NID1 in 1753^p53-/-^cmyc cells was established [Figure 3A]. Cell migration and colony formation assays showed that NID1 KD impaired the migratory and clonogenic abilities of HCC cells [Supplementary Figure 2A and B]. Subsequently, DCs were co-cultured with 1753^p53-/-^cmyc NID1 NC and NID1 KD cells. The induction of DC maturation by NID1 KD cells was significantly suppressed compared with the effect of NID1 NC cells (*P* = 0.0019) [Figure 3B]. EVs were successfully isolated from NID1 NC and NID1 KD cells and characterized by Western blot, TEM, and NTA [Figure 3C-E]. Further flow cytometric analysis showed that NID1 NC EVs induced the expression of the costimulatory molecules CD80 and CD86, as the DC maturation inducer lipopolysaccharide (LPS) did^[23]^, while NID1 KD EVs failed to do so [Figure 3F]. Analysis of the immune regulatory molecules PD-L2, TIM-3, and CD200 showed that NID1 NC EVs drove DCs towards a regulatory state [Supplementary Figure 3 and Figure 3G]. Moreover, CCL17 levels were significantly higher in the supernatant of NID1 NC EV-treated BMDCs than in the NID1 KD EV-treated group (*P* = 0.0014) [Figure 3H].

**Figure 3:**
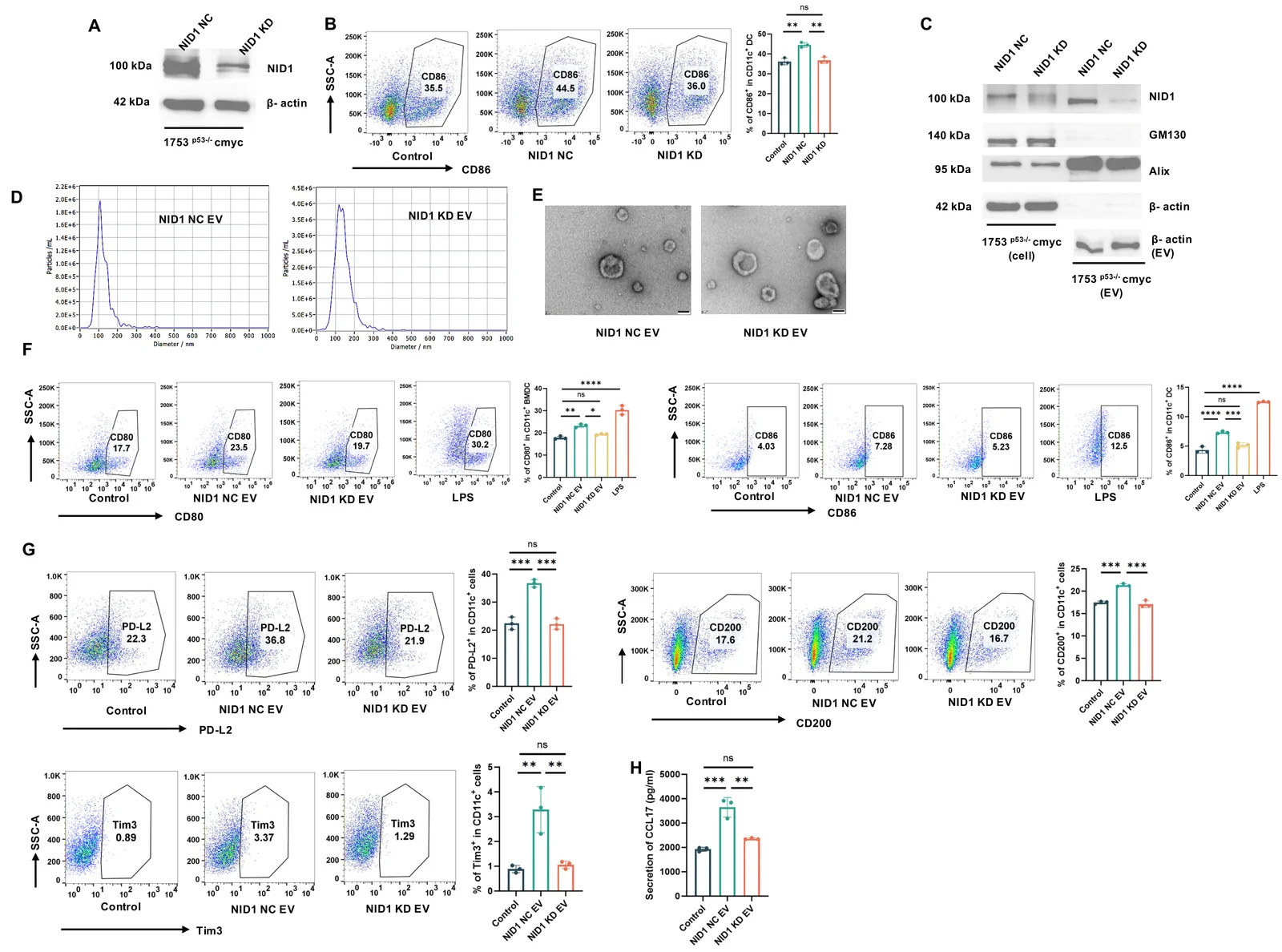
EV-NID1 promotes differentiation of DCs into mregDCs. (A) Western blot demonstration of NID1 expression in 1753^p53-/-^cmyc NID1 NC and NID1 KD cells; (B) Flow cytometric analysis of CD86 expression in BMDCs following co-culture with 1753^p53-/-^cmyc NID1 NC and NID1 KD cells; (C) Detection by Western blot of EV positive marker (Alix), negative marker (GM130), and NID1 expression in cell lysate and EVs of 1753^p53-/-^cmyc NID1 NC and NID1 KD cells; (D) NTA profiles of EVs isolated from 1753^p53-/-^cmyc NID1 NC (left) and NID1 KD (right) cells; (E) TEM imaging of EVs secreted by 1753^p53-/-^cmyc NID1 NC (left) and NID1 KD (right) cells. Scale bar: 100 nm; (F) Flow cytometry analysis of changes in the expression of CD80 and CD86 on BMDCs after treatment with NID1 NC and NID1 KD EVs; (G) Flow cytometry analysis of changes in the expression of PD-L2, TIM-3 and CD200 after treatment with NID1 NC and NID1 KD EVs; (H) ELISA determination of CCL17 levels in BMDC culture supernatants after treatment with NID1 NC or KD EVs (10 μg/mL). Data are presented as the mean ± SD, with statistical significance determined by one-way ANOVA (Tukey’s test). Biological replicates are represented as individual data points. Western blot analysis was performed with three biological replicates. ^*^P < 0.05; ^**^P < 0.01; ^***^P < 0.001; ^****^P < 0.0001. NID1: Nidogen 1; NC: negative control; KD: knockdown; EV: extracellular vesicle; DC: dendritic cell; mregDC: mature regulatory dendritic cell; BMDC: bone marrow-derived dendritic cell; CD80: cluster of differentiation 80; CD86: cluster of differentiation 86; CD200: cluster of differentiation 200; Alix: ALG-2-interacting protein X; NTA: nanoparticle tracking analysis; TEM: transmission electron microscopy; PD-L2: programmed death-ligand 2; TIM-3: T-cell immunoglobulin and mucin-domain containing-3; LPS: lipopolysaccharide; CCL17: C-C motif chemokine ligand 17; ELISA: enzyme-linked immunosorbent assay; SD: standard deviation; ANOVA: analysis of variance; ns: not significant.

A stable NID1 OE cell line was further constructed using NID1-low Hepa1-6 cells [Figure 1D]. EVs were isolated and characterized from NID1 NC and NID1 OE stable clones, respectively [Supplementary Figure 4A-C]. Their capacities to drive DC maturation and induce immunosuppressive markers were then compared. Our results revealed that EVs derived from NID1 OE cells significantly induced DC maturation [Supplementary Figure 4D] and upregulated PD-L2 (*P* < 0.0001), TIM-3 (*P* = 0.0067), and CD200 (*P* = 0.0014) expression [Supplementary Figure 4E], whereas EVs from NID1 NC cells failed to trigger such tolerogenic phenotypes. Collectively, this EV rescue assay provides direct evidence that NID1 packaged within EVs is the primary immunomodulatory cargo responsible for mregDC formation.

### EV-NID1-treated DCs promote the proliferation of CD4^+^ T cells and their differentiation into Tregs

The ability of mregDCs to orchestrate a T helper type 2 (Th2)-polarized response and promote the recruitment of immunosuppressive Tregs contributes to the suppression of antitumor immunity^[24,25]^. After investigating the impact of EV-NID1 on DCs, we further explored the impact of EV-NID1-induced DCs with an mregDC phenotype on T cells. CD4^+^ T cells were isolated from mice and co-cultured with EV-pretreated DCs at a 1:1 ratio [Figure 4A]. DCs treated with NID1 NC EVs significantly promoted the proliferation of CD4^+^ T cells and their differentiation into Tregs (*P* < 0.0001), whereas DCs treated with NID1 KD EVs did not achieve this effect [Figure 4B and C]. These data indicate that EV-NID1-induced mregDCs can induce the generation of Tregs, suggesting that EV-NID1 plays a critical role in establishing an immunosuppressive microenvironment in liver cancer via DCs.

**Figure 4:**
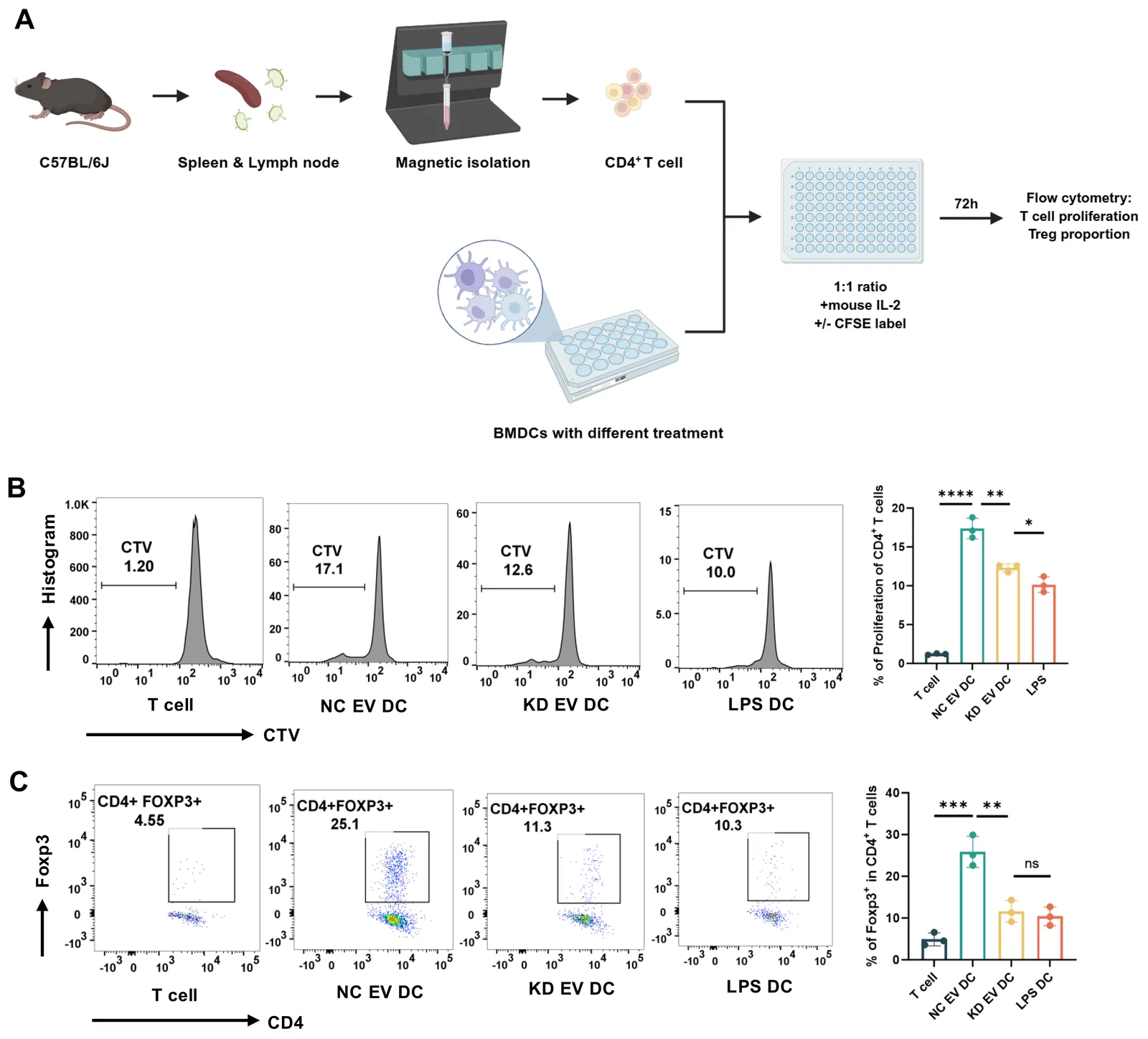
EV-NID1-treated DCs promote the proliferation and Treg differentiation of CD4^+^ T cells. (A) Schematic diagram of the co-culture of CD4^+^ T cells and BMDCs. CD4^+^ T cells isolated by magnetic bead sorting were co-cultured with BMDCs subjected to different treatments (ratio, 1:1), and cells were harvested after 72 h for flow cytometry analysis; (B) Analysis of CD4^+^ T cell proliferation by flow cytometry after co-culture with NID1 NC EV (10 μg/mL), NID1 KD EV (10 μg/mL), or LPS (50 ng/mL); (C) Analysis of Treg proportion in CD4^+^ T cells by flow cytometry after co-culture with NID1 NC EV (10 μg/mL), NID1 KD EV (10 μg/mL), or LPS (50 ng/mL). Data are presented as the mean ± SD, with statistical significance determined by one-way ANOVA (Tukey’s test). Biological replicates are represented as individual data points. ^*^P < 0.05; ^**^P < 0.01; ^***^P < 0.001; ^****^P < 0.0001. NID1: Nidogen 1; NC: negative control; KD: knockdown; EV: extracellular vesicle; DC: dendritic cell; BMDC: bone marrow-derived dendritic cell; CD4: cluster of differentiation 4; Treg: regulatory T cell; FOXP3: forkhead box protein P3; IL-2: interleukin-2; CFSE: carboxyfluorescein succinimidyl ester; CTV: CellTrace Violet; LPS: lipopolysaccharide; SD: standard deviation; ANOVA: analysis of variance; ns: not significant.

### NID1-enriched EVs drive HCC progression via mregDC and Treg accumulation *in vivo*

To validate the physiological relevance of the *in vitro* observations, we established a subcutaneous tumor-bearing mouse model. The results showed that tumors in mice injected with 1753^p53-/-^cmyc NID1 KD cells were significantly smaller than in the NID1 NC group (*P* = 0.0009) [Figure 5A and B]. Flow cytometry analysis revealed that CD4^+^ T cells (*P* = 0.0344) and Treg infiltration (*P* < 0.0001) were significantly lower in the NID1 KD group than in the NID1 NC group [Supplementary Figure 5A and Figure 5C]. Notably, the expression of the immunoregulatory molecules PD-L2 (*P* = 0.0034) and TIM-3 (*P* = 0.0001) was also significantly downregulated on CD11c^+^ DCs in the NID1 KD group [Supplementary Figure 5B and Figure 5D]. To functionally verify that EV-NID1-pulsed DCs are sufficient to restore intratumoral Treg abundance and reverse the growth restriction observed in NID1-deficient tumors, adoptive transfer of BMDCs was performed [Figure 5E]. BMDCs were pre-conditioned with EVs harvested from NID1 NC or NID1 KD tumor cells. Mice bearing NID1 KD subcutaneous tumors received tail vein injections of PBS, NC-EV-pulsed BMDCs, or KD-EV-pulsed BMDCs, respectively. Infusing NC-EV-pulsed DCs partially rescued tumor growth in NID1-KD tumor-bearing mice, whereas KD-EV-pulsed DCs lacked this pro-tumorigenic activity [Figure 5F and G]. Flow cytometry analysis of the dissected tumors suggested that NID1 KD tumors harbored far fewer intratumoral Tregs than NID1 NC tumors. Consistent with the tumor growth rescue phenotype, transfer of NC-EV-pulsed DCs fully restored Treg abundance, an effect not recapitulated by KD-EV-pulsed DCs [Figure 5H]. The indispensable role of Tregs in EV-NID1-mediated HCC progression was further validated via antibody-mediated Treg depletion assays [Supplementary Figure 6A and B]. Notably, Treg depletion substantially slowed tumor progression in NID1 NC tumor-bearing mice, yet still larger than in the NID1 KD group [Supplementary Figure 6C]. This observation aligns with the moderately decreased Treg population in antibody-treated mice, whereas Treg infiltration was profoundly reduced in NID1 KD tumors [Supplementary Figure 6D].

**Figure 5:**
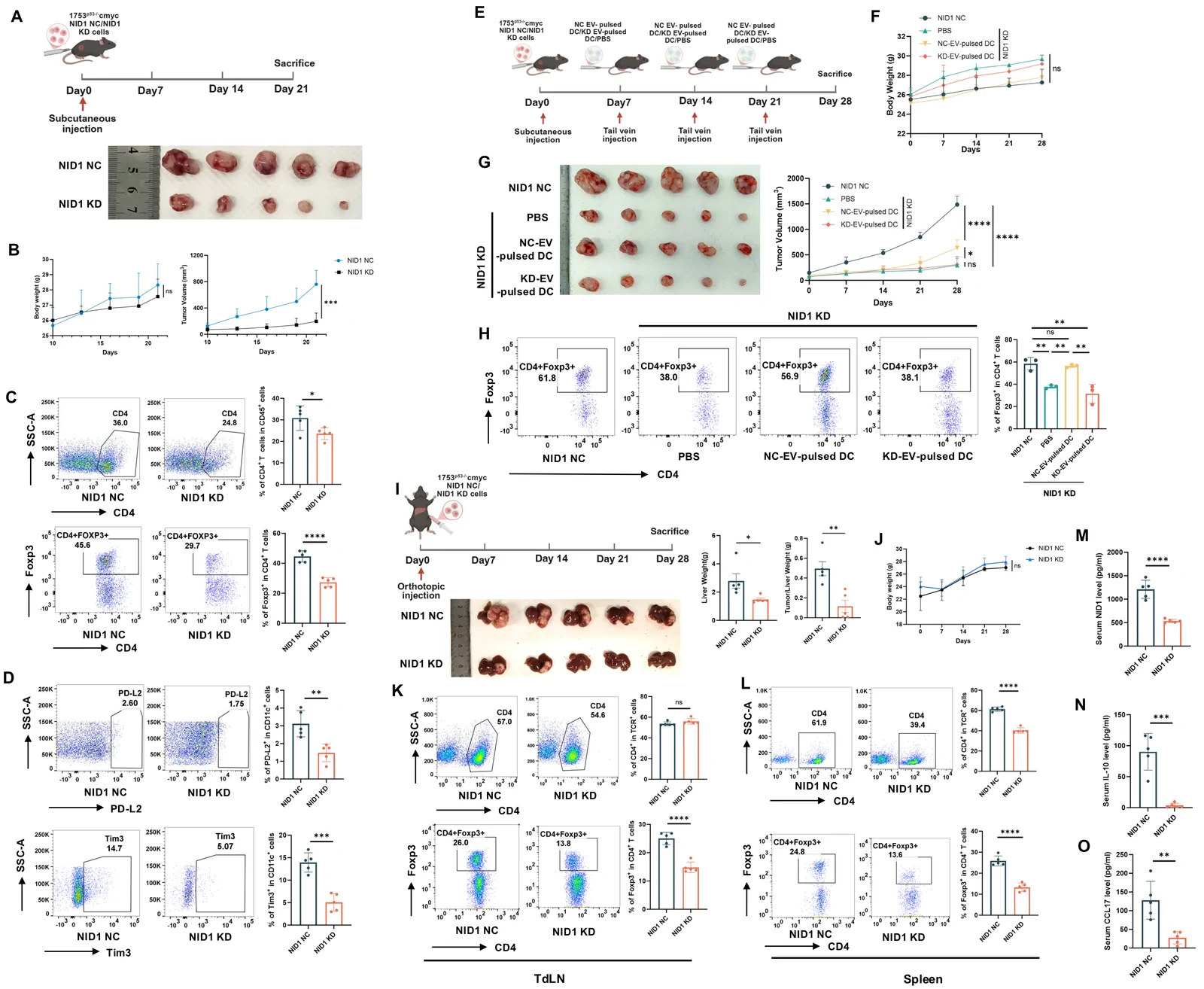
High NID1 expression promotes mregDC and Treg intratumoral infiltration *in vivo*. (A) Flowchart for detecting the subcutaneous growth of 1753^p53-/-^cmyc NID1 NC and NID1 KD cells in mice (upper). 5 × 10^4^ 1753^p53-/-^cmyc NID1 NC/NID1 KD cells were injected into the right dorsal flank of mice (*n* = 5). Tumors were measured every two days. The mice were sacrificed, and tumors were harvested on day 21. Images of isolated subcutaneous tumors (lower); (B) Body weight (left) and tumor volume (right) changes in mice during tumor progression; (C) Flow cytometry analysis of CD4^+^ T cell and Treg infiltration in subcutaneous tumors from NID1 NC and NID1 KD groups (*n* = 5); (D) Flow cytometry analysis of PD-L2 and TIM-3 expression on DCs in subcutaneous tumors from NID1 NC and NID1 KD groups (*n* = 5); (E) Schematic diagram of the DC adoptive transfer experiment. Mice were inoculated with NID1 NC and NID1 KD cells (*n* = 5). Animals bearing NID1 KD tumors received weekly intravenous injections of PBS, NID1 NC-EV-pulsed DCs, or NID1 KD-EV-pulsed DCs for three consecutive weeks. The mice were sacrificed, and tumors were harvested on day 28; (F) Body weight changes in mice during tumor progression; (G) Images of dissected tumors (left) and tumor growth curves (right); (H) Flow cytometry analysis of the Treg proportion within the tumors (*n* = 3); (I) Flowchart for assessing orthotopic hepatic growth of 1753^p53-/-^cmyc NID1 NC and NID1 KD cells in mice (upper). Images of dissected liver tissues on Day 28; (J) Body weight changes in mice during tumor progression. Flow cytometry analysis of CD4^+^ T cell and Treg proportions in TdLNs (K) and spleen (L) from the NID1 NC and NID1 KD groups (*n* = 5); (M-O) ELISA determination of NID1, IL-10, and CCL17 levels in mouse serum from the NID1 NC and NID1 KD groups (*n* = 5). Data are presented as the mean ± SD, with statistical significance determined by one-way ANOVA (multiple groups) (Tukey’s test) or Student’s *t*-test (two groups). Biological replicates are represented as individual data points. Data are presented as the mean ± SD. ^*^P < 0.05; ^**^P < 0.01; ^***^P < 0.001; ^****^P < 0.0001. NID1: Nidogen 1; NC: negative control; KD: knockdown; EV: extracellular vesicle; DC: dendritic cell; mregDC: mature regulatory dendritic cell; PBS: phosphate-buffered saline; CD4: cluster of differentiation 4; FOXP3: forkhead box protein P3; Treg: regulatory T cell; PD-L2: programmed death-ligand 2; TIM-3: T-cell immunoglobulin and mucin-domain containing-3; TdLN: tumor-draining lymph node; IL-10: interleukin-10; CCL17: C-C motif chemokine ligand 17; ELISA: enzyme-linked immunosorbent assay; SD: standard deviation; ANOVA: analysis of variance; ns: not significant.

To further characterize the systemic and regional immune modulatory effects of NID1, an orthotopic implantation model of HCC was adopted. Consistent with subcutaneous tumor findings, NID1 KD significantly inhibited orthotopic HCC tumor growth [Figure 5I and J]. Given that portal and celiac lymph nodes serve as the primary TdLNs for murine hepatic tumors^[26]^, these tissues were collected for further analysis. Flow cytometry showed that despite no significant change in CD4^+^ T cell infiltration within the TdLNs, the infiltration of Tregs was significantly higher in the NID1 NC group compared to the NID1 KD group (*P* < 0.0001) [Figure 5K]. Additionally, flow cytometry analysis of splenocytes showed that infiltration of both CD4^+^ T cells and Tregs was significantly higher in the NID1 NC group than in the NID1 KD group (*P* < 0.0001) [Figure 5L]. These findings suggest that the expression of NID1 in tumors affects activation of the systemic immune system. Serum NID1 levels were reduced in NID1 KD groups, reflecting a corresponding decreased release of NID1 by the NID1 KD tumor [Figure 5M]. Moreover, the serum levels of immunosuppressive cytokines IL-10 (*P* = 0.0002) and CCL17 (*P* = 0.003) were also significantly decreased in mice bearing NID1 KD tumors [Figure 5N and O]. These *in vivo* data support the correlation between high tumoral NID1 levels, larger tumor size, and elevated intratumoral mregDC and Treg infiltration.

## DISCUSSION

EVs have emerged as prominent regulators of the immune response during tumor progression, carrying a diverse array of molecular constituents that serve pivotal roles in immunomodulation^[6]^. Tumor-derived EVs play a dual role in this process, capable of both promoting immune evasion and stimulating an immune response. This functional duality is attributed to their cargo of functional mRNAs^[27]^, non-coding RNAs^[28]^, and proteins^[19]^, which are crucial for interacting with innate and adaptive immunity. PD-L1 present on EVs has been extensively studied for its role in driving tumor immune evasion. It not only directly engages the PD-1/PD-L1 immune checkpoint but also disrupts peripheral T cell homeostasis^[29-31]^. EV-mediated suppression of T cells can also occur through indirect mechanisms. Given that T cell activation depends on co-stimulatory signals from APCs, the indirect modulation of this pathway represents a key mechanism for EV-mediated immunosuppression^[32]^. It has been shown that EVs released by irradiated prostate cancer drive adenosine-mediated suppression of CD8^+^ T cell activation by impairing antigen presentation in DCs^[33]^. In this study, we identified a novel immunosuppressive cascade initiated by HCC-derived NID1-enriched EVs. EV-NID1 rewires iDCs into tolerogenic mregDCs, which subsequently secrete high levels of CCL17 to recruit Foxp3^+^ Tregs and suppress anti-tumor surveillance.

NID1, a glycoprotein component of the basement membrane and ECM, plays a multifaceted role in cancer progression^[34,35]^. It promotes tumorigenesis and metastasis by enhancing cancer cell proliferation, migration, invasion, and angiogenesis, as established across various cancer types^[17]^. However, the immunomodulatory activities of NID1, especially within primary liver cancer lesions, remain poorly characterized, with conflicting reports from non-hepatic disease models. In a myocardial infarction model, NID1 orchestrates a shift in immune cell populations by promoting Treg infiltration and reducing both proinflammatory monocytes/macrophages and cardiac CD8^+^ T cells^[36]^. Another study revealed that NID1 drives macrophage polarization toward an M2-like phenotype, promotes the activation of CD4^+^ T cells, and inhibits the secretion of the pro-inflammatory cytokines tumor necrosis factor-alpha (TNF-α) and interferon-gamma (IFN-γ)^[37]^. Intriguingly, a study in the context of cancer demonstrated that NID1 can drive macrophages toward an M1 phenotype, thereby suppressing tumor growth^[38]^. These divergent results indicate that the immunoregulatory functions of NID1 are pleiotropic and require more extensive study across different diseases. Our findings show that EV-NID1 facilitates HCC progression by reprogramming DCs into an immunosuppressive mregDC phenotype, thus unveiling a new mechanism of NID1-mediated immunomodulation in the tumor microenvironment. Notably, we detected a lower-molecular-weight NID1 variant in EV preparations relative to whole-cell lysates, suggesting that NID1 undergoes proteolytic cleavage prior to being sorted into EVs [Supplementary Figure 7]. This observation is consistent with previous reports suggesting that proteolytic cleavage of NID1 may generate bioactive truncated variants capable of promoting cell invasion and tissue penetration^[39,40]^. Future research dissecting the protease cascades regulating NID1 truncation and the EV sorting machinery responsible for packaging cleaved NID1 will clarify how distinct NID1 variants exert divergent effects in HCC. Moreover, several studies have shown that tumor antigens associated with EVs are taken up more efficiently by immune cells than their soluble counterparts^[41-43]^. Our findings further support a greater effect of the EV-associated NID1 form over the secreted NID1 form in inducing DC maturation and subsequent differentiation to mregDCs, pointing to its critical function in tumor immunoregulation. The current study only completes phenotypic and causal validation of the EV-NID1 immune axis; upstream DC surface receptors and intracellular signaling pathways activated by EV-NID1 remain uncharacterized and will be explored in follow-up investigations.

The novel DC state, mregDC, is characterized by its dual capacity to generate immunostimulatory and immunoregulatory molecules^[25]^. mregDCs are defined by their unique capacity to produce immunoregulatory molecules, a stark contrast to other DCs. Their profile includes high levels of maturation markers (CD40, CD80, CCR7) alongside immunoregulatory molecules such as PD-L1, PD-L2, and TIM-3^[44]^. The present study demonstrates that EV-NID1 can promote the expression of CD80, CD86, MHC II, and the immunoregulatory molecules PD-L2 and TIM-3 in DCs. However, an upregulation of PD-L1 was not observed in our study [Supplementary Figure 8], possibly masked by its high constitutive expression in DCs. PD-L1 is expressed on various immune cells and tumor cells, whereas PD-L2 expression is primarily restricted to professional APCs such as activated DCs^[45]^. We therefore hypothesize that PD-L2 plays a pivotal role in shaping the immunosuppressive microenvironment within the EV-NID1-induced mregDC phenotype. Accumulating evidence indicates that mregDCs represent the predominant cellular source of CCL17 within the tumor immune microenvironment, and this chemokine acts as a critical paracrine signal that specifically drives the chemotactic recruitment of circulating Foxp3^+^ Tregs into tumor lesions^[46]^. CCL17 thus serves as the core molecular bridge linking mregDC polarization to robust intratumoral Treg accumulation, which ultimately establishes a profoundly immunosuppressive niche that blunts anti-tumor immunity. Consistent with this established regulatory axis, our *in vitro* experiments showed that EV-NID1 promotes CCL17 secretion by DCs. Together with our *in vivo* finding that serum CCL17 levels were decreased in mice injected with NID1 KD cells, this points to a potential role for EV-NID1 in reshaping DCs toward a tolerogenic mregDC phenotype. Here, mregDC-derived CCL17 functions as the key chemokine mediator to orchestrate Treg recruitment and expansion within HCC tissues.

Several limitations of the present work need to be acknowledged to inform follow-up mechanistic and translational studies. In the present study, the functional immune data were generated using murine HCC cell lines and syngeneic mouse models. Given the differences between mice and humans in DC subtypes, Treg signaling pathways, and ECM-EV interactions, the defined correlation between EV-NID1 and the mregDC/Treg regulatory axis cannot be directly translated to human HCC patients. Additional testing using liver tumor clinical samples, patient-circulating EVs, and primary human DC/T cell co-cultures is needed to confirm these findings.

Furthermore, this study only characterized the downstream immune phenotypes induced by EV-NID1. The upstream molecular processes, such as DC surface receptors that bind EV-NID1 and the corresponding intracellular signaling pathways, remain unexamined. Additionally, differential ultracentrifugation alone cannot completely exclude co-isolated non-specific soluble protein aggregates or other co-sedimented contaminants alongside EV pellets. Although our immuno-gold TEM data showed that NID1 predominantly localizes to the outer lipid bilayer of EVs rather than accumulating within the EV^[47]^, the specificity of the EV-associated pro-tumorigenic effect of NID1 still requires further verification. Moreover, the present study focused on CCL17 as the major Treg chemoattractant released by EV-NID1-treated mregDCs and did not include a full multi-omics analysis to profile all tolerogenic factors secreted by these DCs. Follow-up transcriptomic and proteomic work will help comprehensively identify other immunosuppressive cytokines and chemokines produced by EV-NID1-modified DCs.

With this mechanistic framework established, future preclinical work may develop NID1-targeted silencing or EV-NID1 neutralizing strategies to disrupt tumor-originated immunosuppressive cascades and boost the response to HCC immunotherapy^[48,49]^.

In conclusion, our study supports a working model in which HCC-derived, NID1-enriched EVs promote tumor progression by driving the phenotypic conversion of DCs into a regulatory phenotype (mregDCs) and facilitating Treg expansion.

## DECLARATIONS

### Availability of data and materials

The data that support the findings of this study are available from the corresponding author upon reasonable request.

### Copyright

© The Author(s) 2026.
