# Supplementary material for: Nidogen 1-enriched extracellular vesicles drive HCC progression by inducing mregDC differentiation and Treg infiltration
Source: Extracell Vesicles Circ Nucl Acids. 2026 Sep 8;7(3):1411–27. doi: 10.20517/evcna.2026.03 (PMC13673350; doi:10.20517/evcna.2026.03)
Supplement: Supplementary file 1 [file evcna-7-3-1411-SupplementaryMaterials.pdf]

Supplementary Materials

Nidogen 1-enriched extracellular vesicles drive HCC progression by inducing mregDC differentiation and Treg infiltration

Lu Xia[1,#], Tingmao Xue[1,#], Yining Xu[1], Judy Wai Ping Yam[2,3,4], Xiaowen Mao[1]

[1]State Key Laboratory of Mechanism and Quality of Chinese Medicine, Institute of Chinese Medical Sciences, University of Macau, Macao 999078, China.
[2]Department of Pathology, Li Ka Shing Faculty of Medicine, The University of Hong Kong, Hong Kong SAR, China.
[3]State Key Laboratory of Liver Research (The University of Hong Kong), Hong Kong SAR, China.
[4]Materials Innovation Institute for Life Sciences and Energy (MILES), HKU-SIRI, Shenzhen 518000, Guangdong, China.
[#]These authors contributed equally to this work.

Correspondence to: Dr. Xiaowen Mao, State Key Laboratory of Mechanism and Quality of Chinese Medicine, Institute of Chinese Medical Sciences, University of Macau, Macao 999078, China. E-mail: susanmao@um.edu.mo

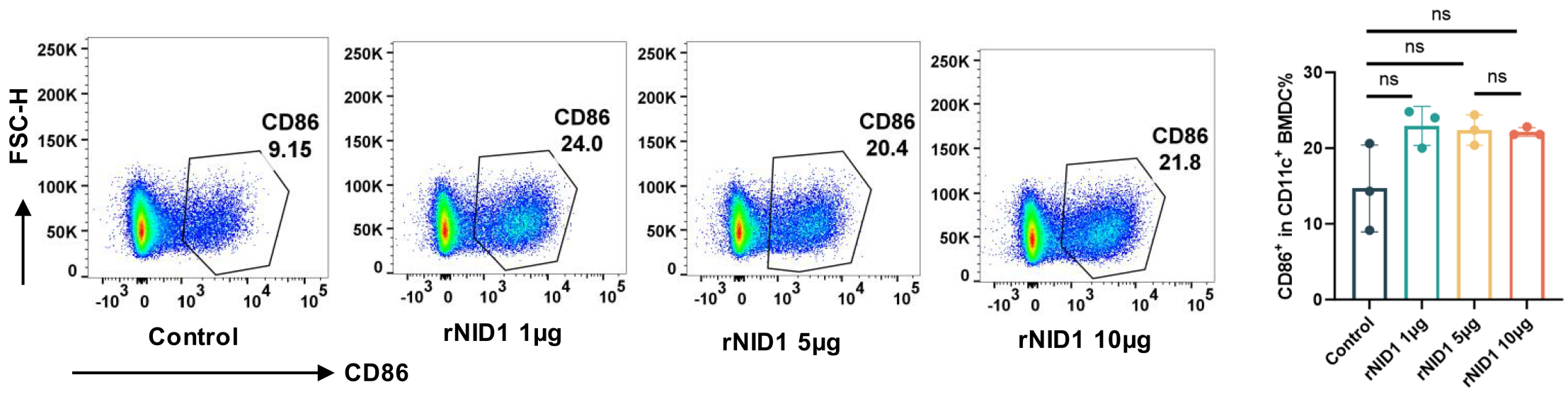

Supplementary Figure 1. rNID1 is unable to induce DC maturation. Flow cytometric analysis of CD86 expression in BMDCs following treatment with different concentrations (1, 5, 10 μg/mL) of recombinant NID1. Data are presented as the mean ± SD, with statistical significance determined by one-way ANOVA (Tukey's test). Biological replicates are represented as individual data points. ns, not significant.

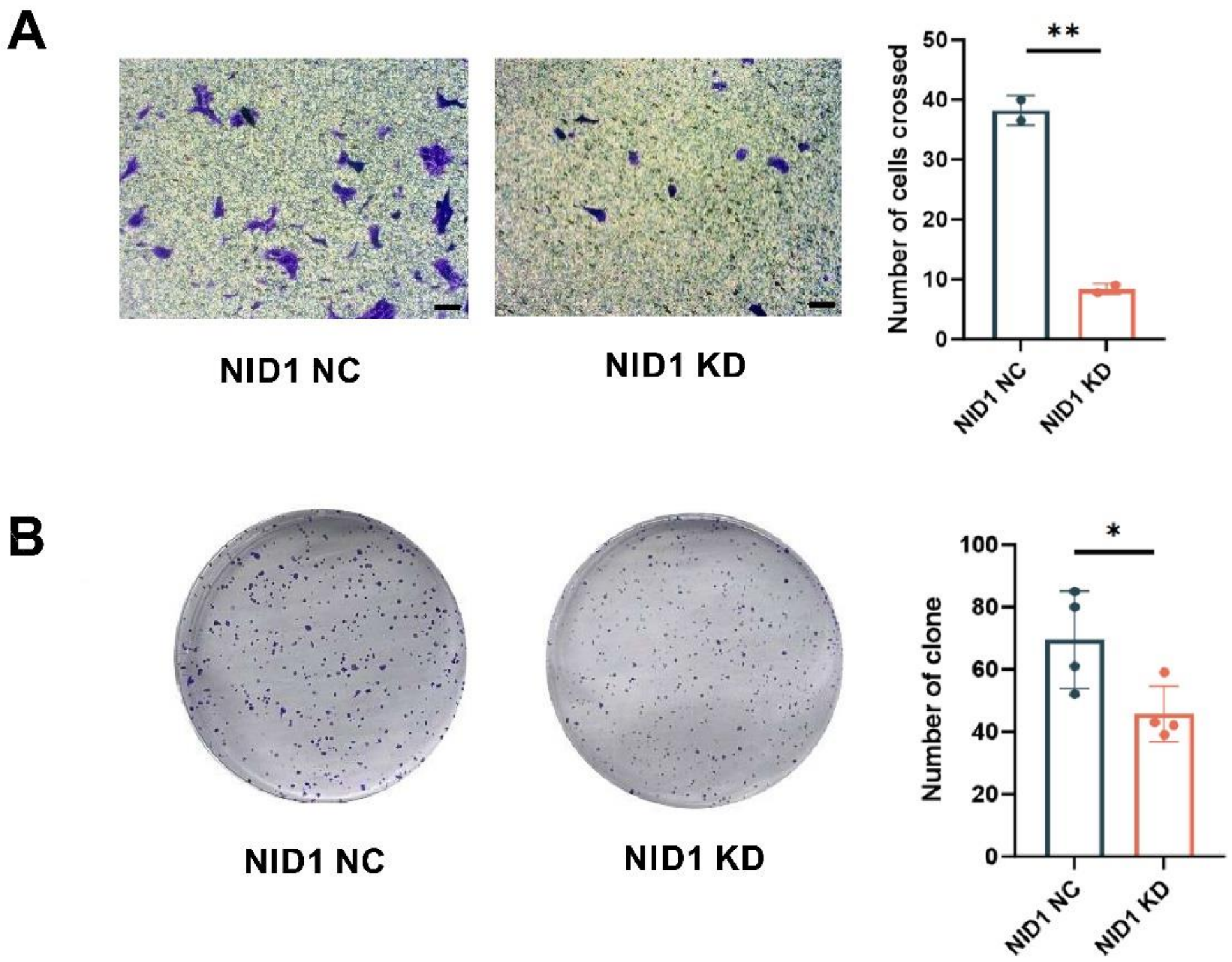

Supplementary Figure 2. NID1 promotes the migratory and clonogenic abilities of liver cancer cells. (A) Cell migration was evaluated by a Transwell migration assay in $1753^{p53-/-}$cmyc NID1 NC and NID1 KD cell lines. Scale bar: 50μm; (B) A clone formation assay was used to examine the clone formation ability of $1753^{p53-/-}$cmyc NID1 NC and NID1 KD cell lines. Data are presented as the mean ± SD, with statistical significance determined by Student's *t*-test. Biological replicates are represented as individual data points. *$P < 0.05$; **$P < 0.01$ from Student's *t*-test.

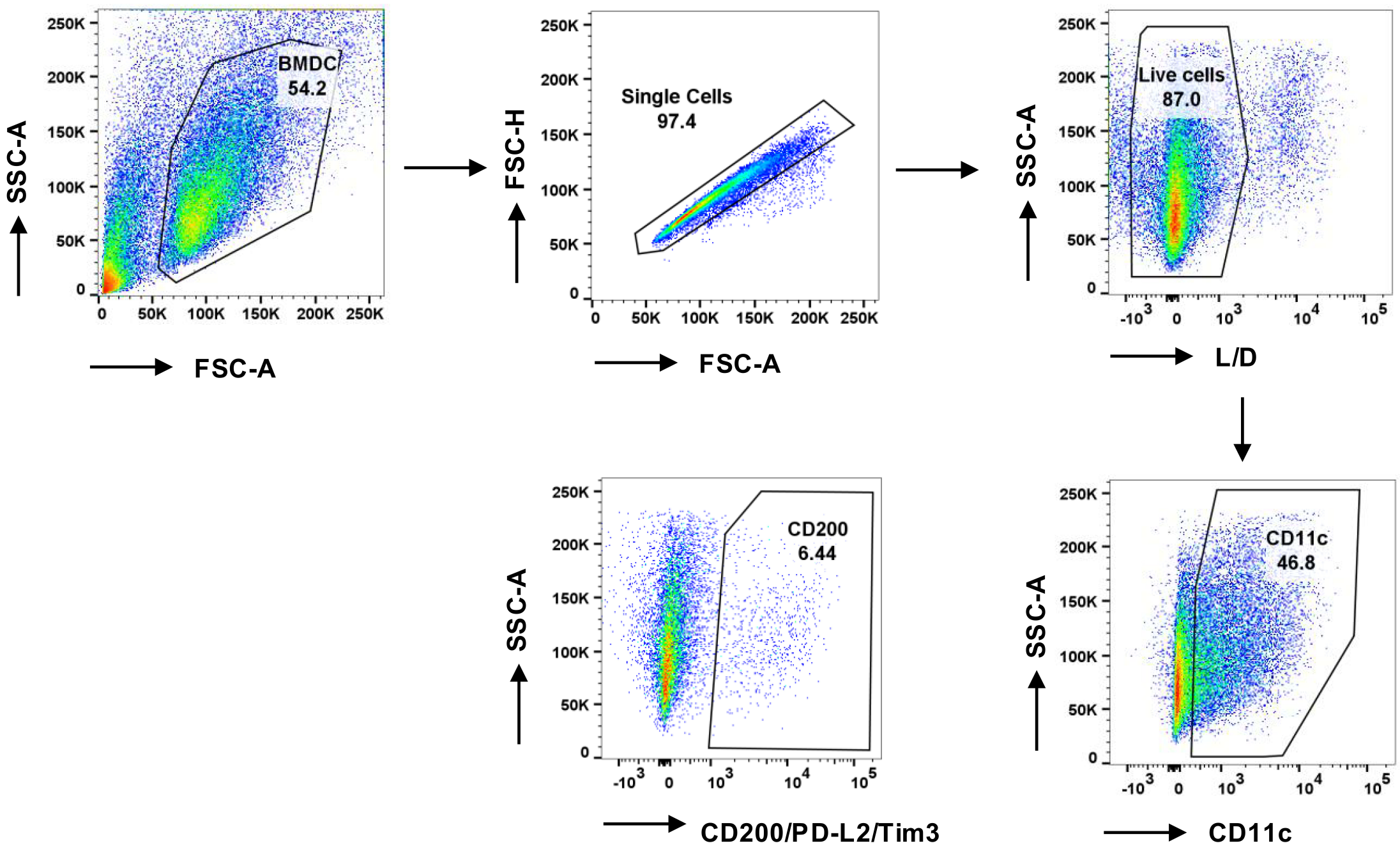

Supplementary Figure 3. Flow cytometry gating strategy for the expression of immunoregulatory molecules (PD-L2, Tim-3, CD200) on BMDCs.

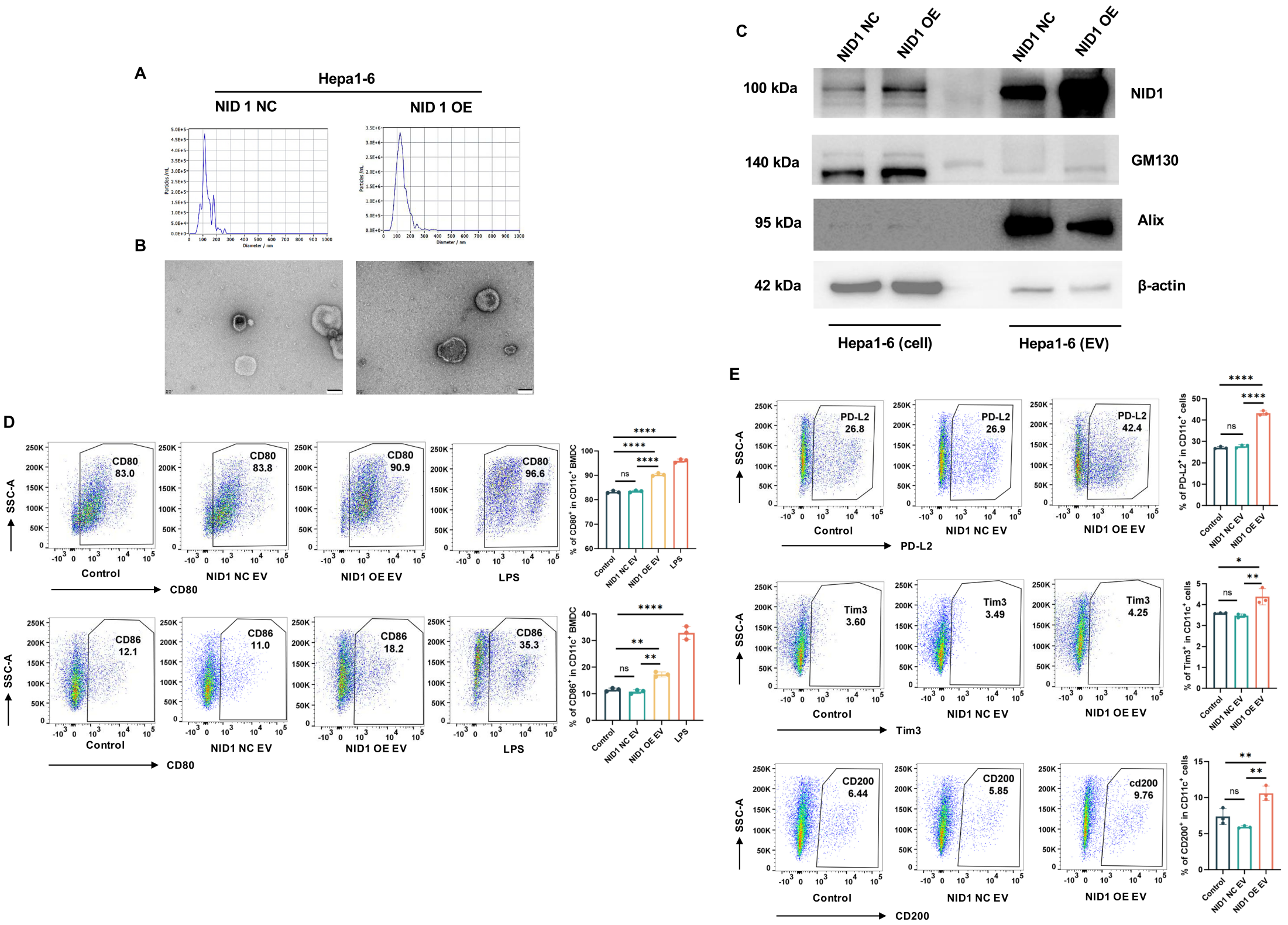

Supplementary Figure 4. NID1-overexpressing EVs promote the maturation of DCs and polarize DCs into mregDCs. (A and B) NTA profiles and TEM images of EVs isolated from Hepa1-6 NID1 NC and NID1 OE cells. Scale bar: 100 nm; (C) Western blot analysis of the EV positive marker (Alix), EV negative marker (GM130), and NID1 expression in cell lysate and EVs of Hepa1-6 NID1 NC and NID1 OE cells; (D) Flow cytometry analysis of changes in the expression of CD80 and CD86 on BMDCs after treatment with NID1 NC and NID1 OE EVs; (E) Flow cytometry analysis of changes in the expression of PD-L2, Tim-3, and CD200 after treatment with NID1 NC and NID1 OE EVs. Data are presented as the mean ± SD, with statistical significance determined by one-way ANOVA (Tukey's test). Biological replicates are represented as individual data pointsWestern blot analysis was performed with three biological replicates. *$P < 0.05$; **$P < 0.01$; ***$P < 0.001$; ****$P < 0.0001$; ns, not significant.

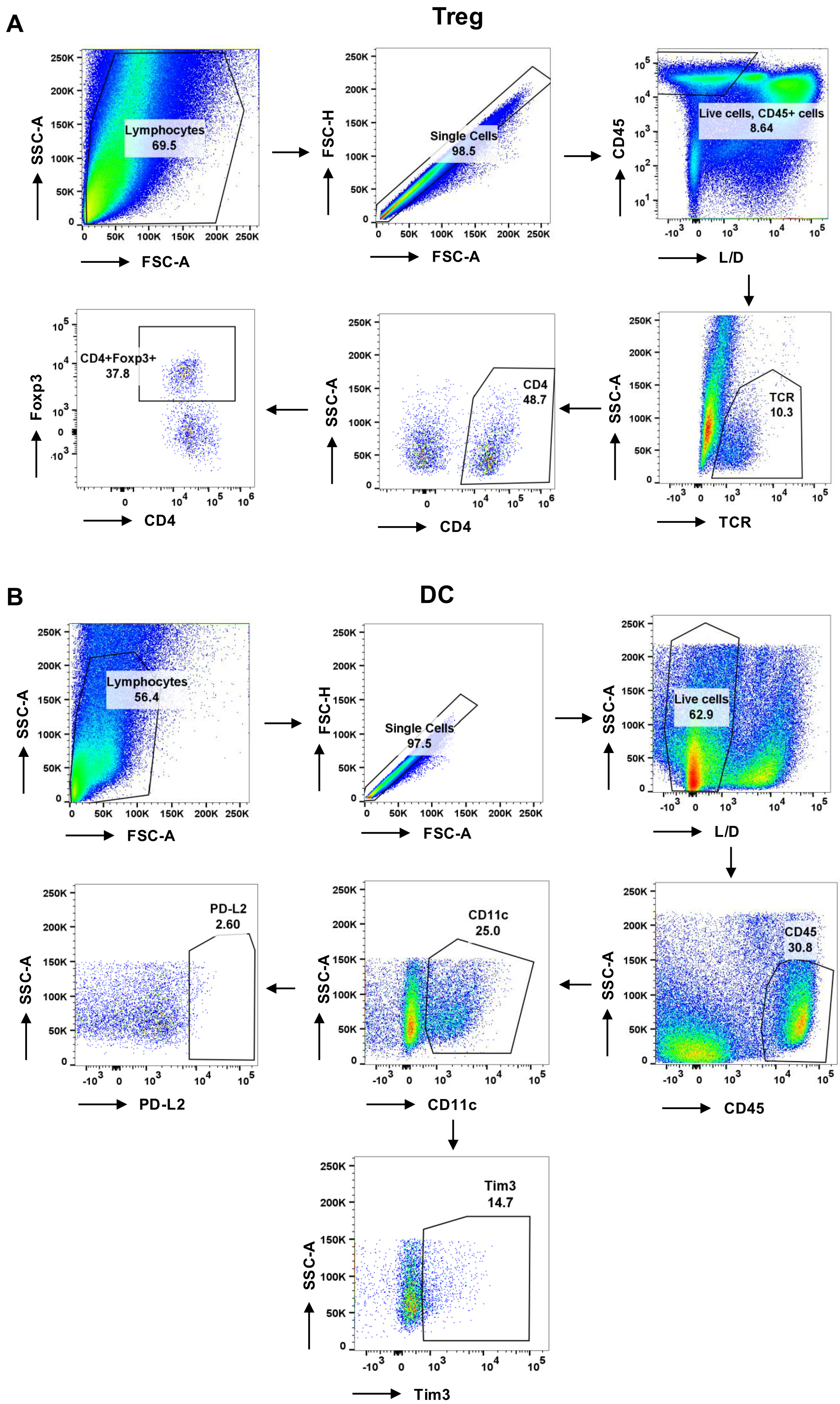

Supplementary Figure 5. Flow cytometry gating strategy for Treg ($CD45^{+}TCR^{+}CD4^{+}FOXP3^{+}$) and DC ($CD45^{+}CD11c^{+}$) in mouse tumor tissues. (A) Treg; (B) DC.

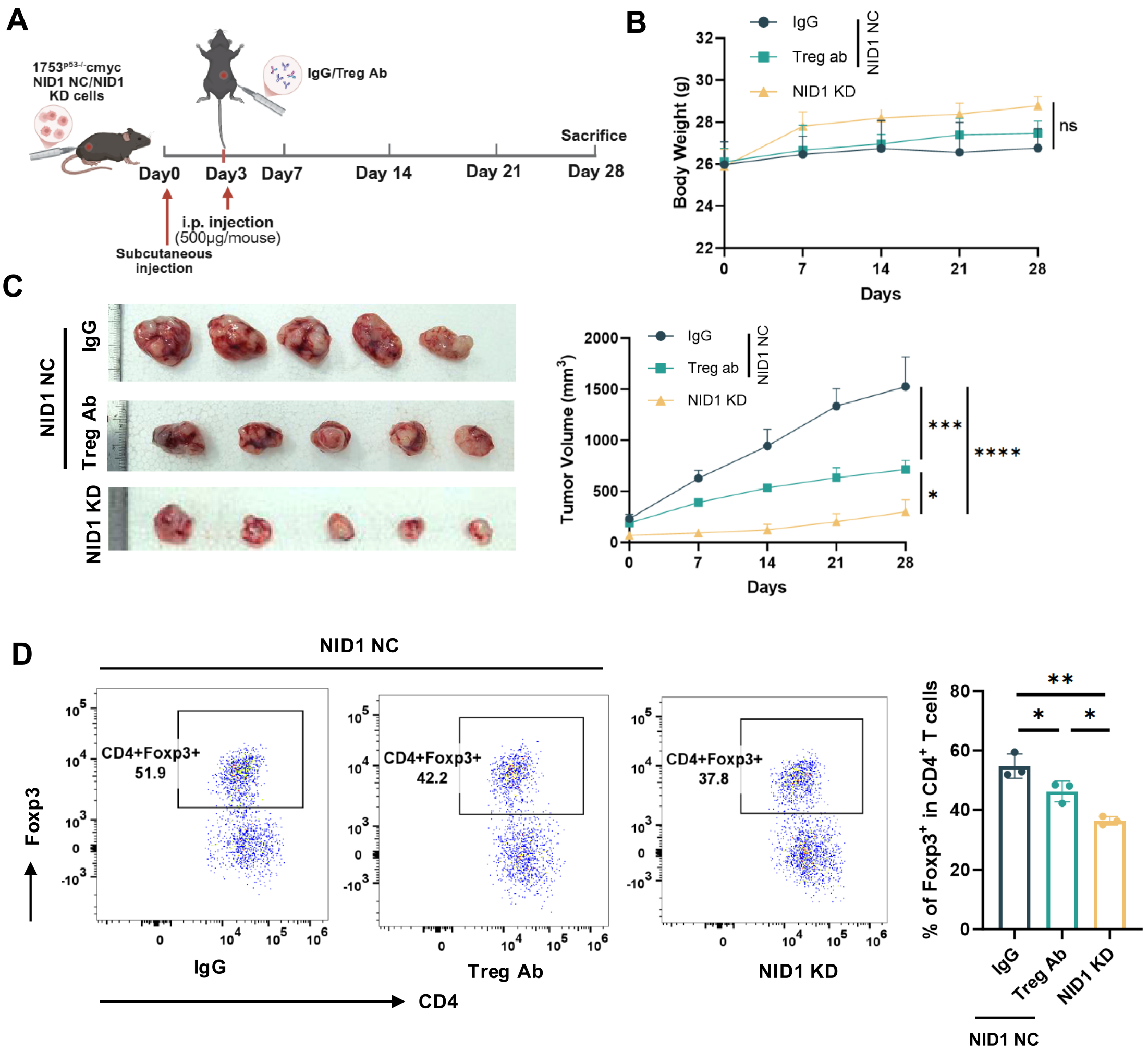

Supplementary Figure 6. Treg depletion assay reveals an indispensable role of Tregs in NID1-driven HCC growth. (A) Schematic diagram of the Treg depletion experiment. Mice were inoculated with NID1 NC and NID1 KD cells and received intraperitoneal injections of IgG and anti-Treg antibody, respectively (n=5); (B) Body weight changes in mice during tumor progression; (C) Images of dissected tumors (left) and tumor growth (right) curves; (D) Flow cytometry analysis of the Treg proportion within the tumor ($n = 3$). Data are presented as the mean ± SD, with statistical significance determined by one-way ANOVA (Tukey's test). *$P < 0.05$; **$P < 0.01$; ***$P < 0.001$; ****$P < 0.0001$; ns, not significant.

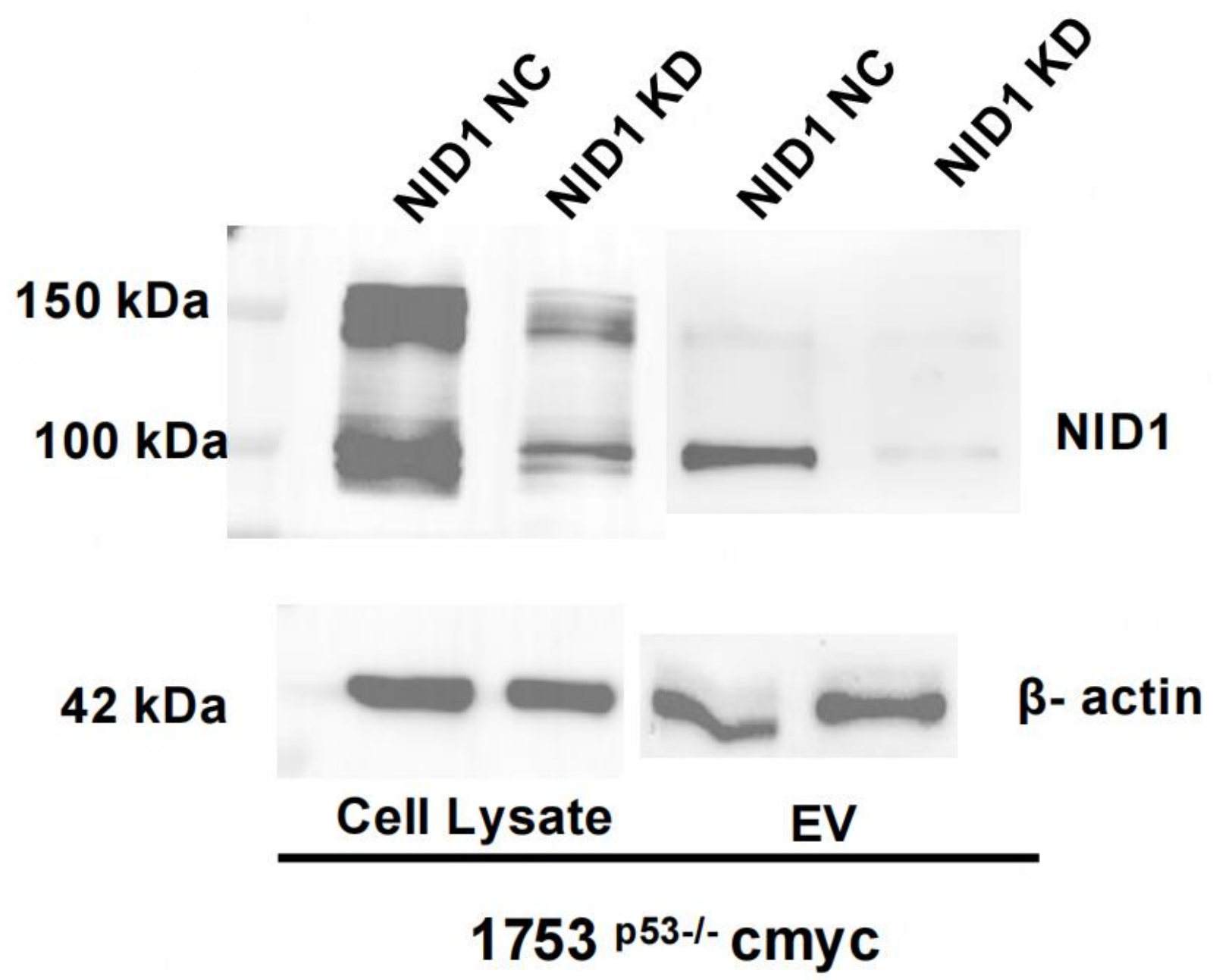

Supplementary Figure 7. Truncated NID1 is incorporated into EVs. Expression of NID1 variants of different molecular weights in total cell lysates and isolated EVs from NID1 NC and NID1 KD cells. Western blot analysis was performed with three biological replicates.

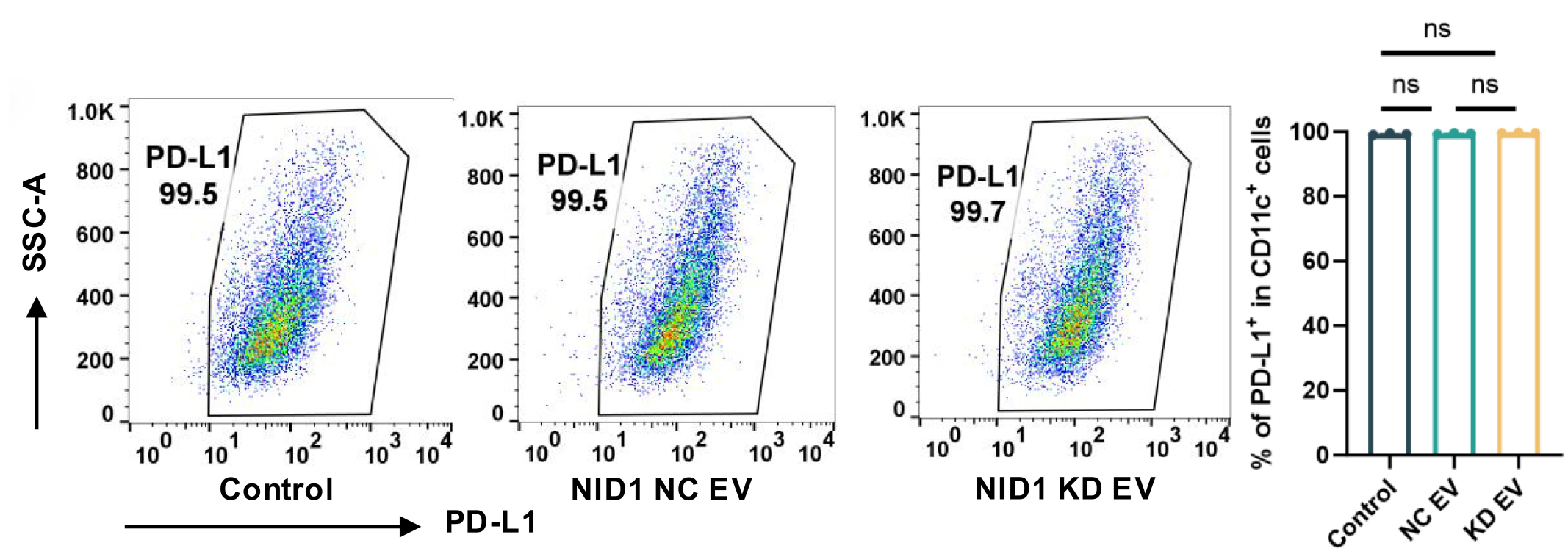

Supplementary Figure 8. NID1 EVs are unable to induce PD-L1 expression in DCs. (A) Flow cytometry analysis of changes in PD-L1 on BMDCs after treatment with NID1 NC EVs and NID1 KD EVs. Data are presented as the mean ± SD, with statistical significance determined by one-way ANOVA (Tukey's test). Biological replicates are represented as individual data points. ns, not significant.
